# First Global‐Scale Synoptic Imaging of Solar Eclipse Effects in the Thermosphere

**DOI:** 10.1029/2020JA027789

**Published:** 2020-09-18

**Authors:** Saurav Aryal, J. S. Evans, John Correira, Alan G. Burns, Wenbin Wang, Stanley C. Solomon, Fazlul I. Laskar, William E. McClintock, Richard W. Eastes, Tong Dang, Jiuhou Lei, Huixin Liu, Geonhwa Jee

**Affiliations:** ^1^ Labrotary for Atmospheric and Space Physics University of Colorado Boulder CO USA; ^2^ Computational Physics Inc Springfield VA USA; ^3^ High Altitude Observatory National Center for Atmospheric Research Boulder CO USA; ^4^ CAS Key Laboratory of Geospace Environment, School of Earth and Space Sciences University of Science and Technology of China Hefei China; ^5^ Department of Earth and Planetary Sciences Kyushu University Fukuoka Japan; ^6^ Division of Polar Climate Sciences Korea Polar Research Institute Incheon South Korea

## Abstract

A total solar eclipse occurred in the Southern Hemisphere on 2 July 2019 from approximately 17 to 22 UT. Its effect in the thermosphere over South America was imaged from geostationary orbit by NASA's Global‐scale Observation of Limb and Disk (GOLD) instrument. GOLD observed a large brightness reduction (>80% around totality) in OI 135.6 nm and N_2_ LBH band emissions compared to baseline measurements made 2 days prior. In addition, a significant enhancement (with respect to the baseline) in the ΣO/N_2_ column density ratio (~80%) was observed within the eclipse's totality. This enhancement suggests that the eclipse induced compositional changes in the thermosphere. After the eclipse passed, a slight enhancement in ΣO/N_2_ column density ratio (~7%) was also seen around the totality path when compared to measurements before the eclipse. These observations are the first synoptic imaging measurements of an eclipse's thermospheric effects with the potential to drastically improve and test our understanding of how the thermosphere responds to rapid, localized changes in solar short wavelength radiation.

## Key Points

- First synoptic global‐scale observations of a solar eclipse's effect on the thermosphere are presented based on FUV imaging
- Greater than 80% reduction around eclipse totality is seen in OI 135.6 nm and N_2_ LBH emission brightnesses
- Enhanced ΣO/N_2_ column density ratio (~80%) is observed within eclipse's totality and after the eclipse (~7%) suggesting compositional changes

## 1 Introduction

During a solar eclipse, the Moon's shadow traverses the Earth's atmosphere at supersonic speeds. The obscuration of high‐energy solar radiation (ultraviolet and X‐rays) in the eclipse's shadow causes a localized, rapid reduction in ionization and heating in the upper atmosphere. Eclipse‐induced cooling of the atmosphere creates a pressure gradient resulting in winds converging toward the totality region. This flow alters the dynamics of the entire Ionosphere‐Thermosphere (IT) system. In addition, a reduction in ionization leads to changes in the photochemistry of the IT system. Furthermore, the spatially confined nature of solar radiation obscuration and quick recovery back to daytime conditions creates a unique forcing within the IT system. Thus, observing a solar eclipse's effects on the IT system provides a unique natural experiment for testing our understanding of physical processes there.

The effects of an eclipse on the IT system have been observed previously. Most observations have been of ionospheric parameters (Coster et al., 2017; Davis & Rosa, 1970; Ledig et al., 1946; Mrak et al., 2018, etc.). Ledig et al. (1946) presented measurements of reduced electron number density over Huancayo, Peru, during the 1946 solar eclipse. A few decades later, Davis and Rosa (1970) reported wave‐like perturbations in the column electron density during the 1970 eclipse. Chimonas and Hines (1971) explained that those fluctuations result from atmospheric gravity waves (AGWs) propagating into the thermosphere. Chimonas and Hines (1970) predicted that eclipses induce AGWs as a result of pressure and temperature gradients in a gravitationally stratified atmosphere. At IT altitudes, these AGWs are expected to create ionospheric waves via collisional coupling between the thermosphere and the ionosphere.

In contrast to the numerous ionospheric observations of an eclipse's effect, thermospheric observations are rare. Combined thermospheric and ionospheric measurements were made by the Low Earth Orbiting (LEO) Challenging Minisatellite Payload (CHAMP) satellite during the 8 April 2005 solar eclipse near totality (Tomás et al., 2007). But they found no clear thermospheric change due to the eclipse. This was most likely because only a “snapshot” of the eclipse was observed due to CHAMP's orbital track. During the 2017 solar eclipse over the continental USA, Harding et al. (2018) observed wave‐like neutral wind perturbations far away from the eclipse's path. Their result was based on Fabry Perot Interferometer (FPI) measurements of the nighttime OI 630.0 nm (red line, peak height 250 km) brightness. For the same eclipse, Aryal et al. (2019) concluded that the enhanced nighttime red‐line brightness they observed over Carbondale, IL (in the path of totality), hours after the eclipse's end, was most likely due to a persistent enhancement of the atomic oxygen density induced by the eclipse.

Regular nighttime thermospheric measurements are made using ground‐based optical instruments, such as FPIs. However, most ground‐based optical instruments do not operate during the daytime because the solar background contribution is high, even during solar eclipses. UV measurements from LEO satellites are not affected by the daytime solar background. However, the orbital trajectory of LEO satellites restricts where the measurements can be made and thus reduces the likelihood of coincidences with totality. NASA's Global‐scale Observation of Limb and Disk (GOLD; Eastes et al., 2017) instrument in a geostationary orbit, in contrast, can provide synoptic imaging of eclipse's effects on the thermosphere. GOLD is a FUV spectrograph that was launched in 2018 onboard the SES‐14 satellite, now located above Brazil (47.5°W longitude). GOLD observations have already enabled synoptic global‐scale study of the IT system (Eastes et al., 2019; Gan et al., 2020a, 2020b; Karan et al., 2020). Please refer to Eastes et al. (2020) for details on initial GOLD observational results and their significance.

In this paper, we present the first global‐scale imaging of thermospheric effects during an eclipse. The images are presented as brightness depletions caused by the eclipse's shadow on two prominent upper atmospheric airglow emissions: the O I 135.6 nm and the N_2_ Lyman‐Bridge‐Hopfield (LBH) band system. We use these emission brightnesses to analyze the ΣO/N_2_ column density ratio. This ratio is an indicator of thermospheric compositional changes (Correira et al., 2020; Evans et al., 1995; Strickland et al., 1995). Future data‐model comparisons of GOLD's global‐scale eclipse observations (and other supplementary measurements) with various IT models will enable us to better understand the IT system's response to localized, impulsive drivers.

## 2 GOLD Observations of the 2019 Solar Eclipse

GOLD is a FUV imaging spectrometer (~133–165 nm) with three interchangeable entrance slits that can be selected to achieve different resolutions (0.21, 0.35, or 2.16 nm). GOLD also has two identical channels, each with its own mirror that can independently scan the Earth from a geostationary orbit above 47.5°W longitude. These mirrors are used to reflect light into one of the three entrance slits where the light gets dispersed. The spectral and spatial information of the dispersed light is then recorded and reduced (Eastes et al., 2017).

**Figure 1:**
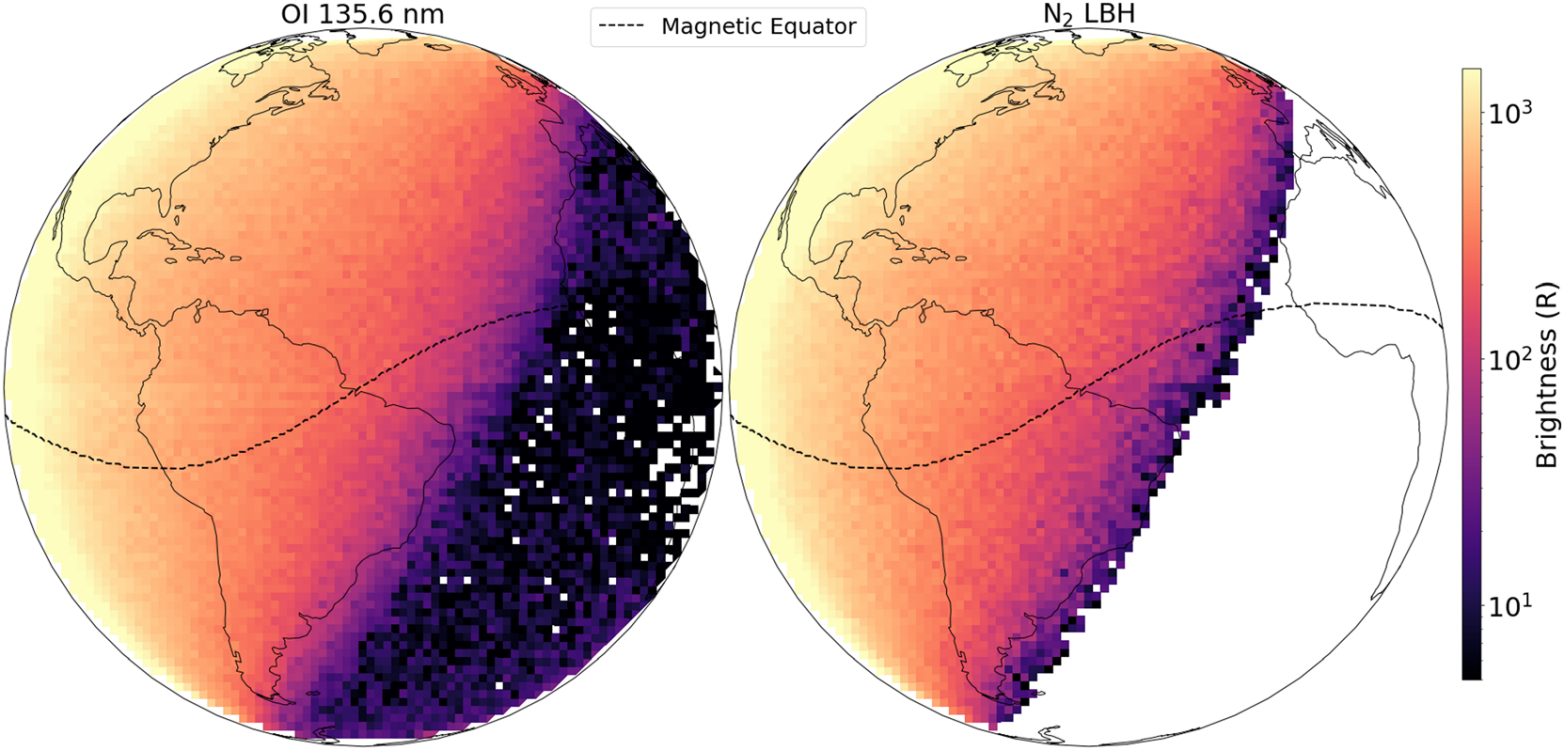
Typical GOLD imaging of the O I 135.6 nm (left) and N_2_ LBH band (right) emission brightness (logarithmic scale) on 30 June 2019. The northern scans were made at a cadence of ~15 min starting at 22:10 UT on the northeastern edge of the disk and ending on the northwest edge of the disk. The southern scans were made at the same cadence starting at 22:22 UT on the southeast and ending on the southwest edge of the disk. The northern and southern scans are combined to get the full disk image at a cadence of ~30 min. The location of the magnetic equator (dashed‐black line) and continental outlines have been added for reference. Since there is no nighttime source of LBH emission, it is well below the noise level and has been removed. Thus, the white space on LBH brightness image (right) represents the nightside. The white pixels seen in the 135.6 nm nightside images are negative values encountered during background subtraction.

GOLD makes daytime disk, limb, occultation, and nighttime disk measurements at different cadences. For this study, we used day disk imaging of the OI 135.6 nm and N_2_ LBH band emission brightnesses. The full disk day images were generated by scanning east to west starting from the northeastern limb and ending at the southwestern limb. Northern and Southern Hemisphere scans were made separately (~15 min cadence each) at a spectral dispersion of 0.04 nm/pixel. Data counts were then converted to radiance in Rayleighs (R) per nanometer. Integrating the radiance over the desired wavelength range gives the brightness in *R* (1*R* = 10^10^ photons m^−2^ s^−1^ steradian^−1^). Figure 1 shows an example of typical daytime 135.6 nm and LBH brightness imaging. Integration from 134 to 137 nm for the 135.6 nm emission and from 140 to 160 nm (excluding the N I 149.1 nm feature) for the LBH band emission was used to calculate the brightnesses. The scans in the Northern Hemisphere were made starting at 22:10 UT, while the southern scan started at 22:22 UT on 30 June 2019 (total cadence ~30 min).

**Figure 2:**
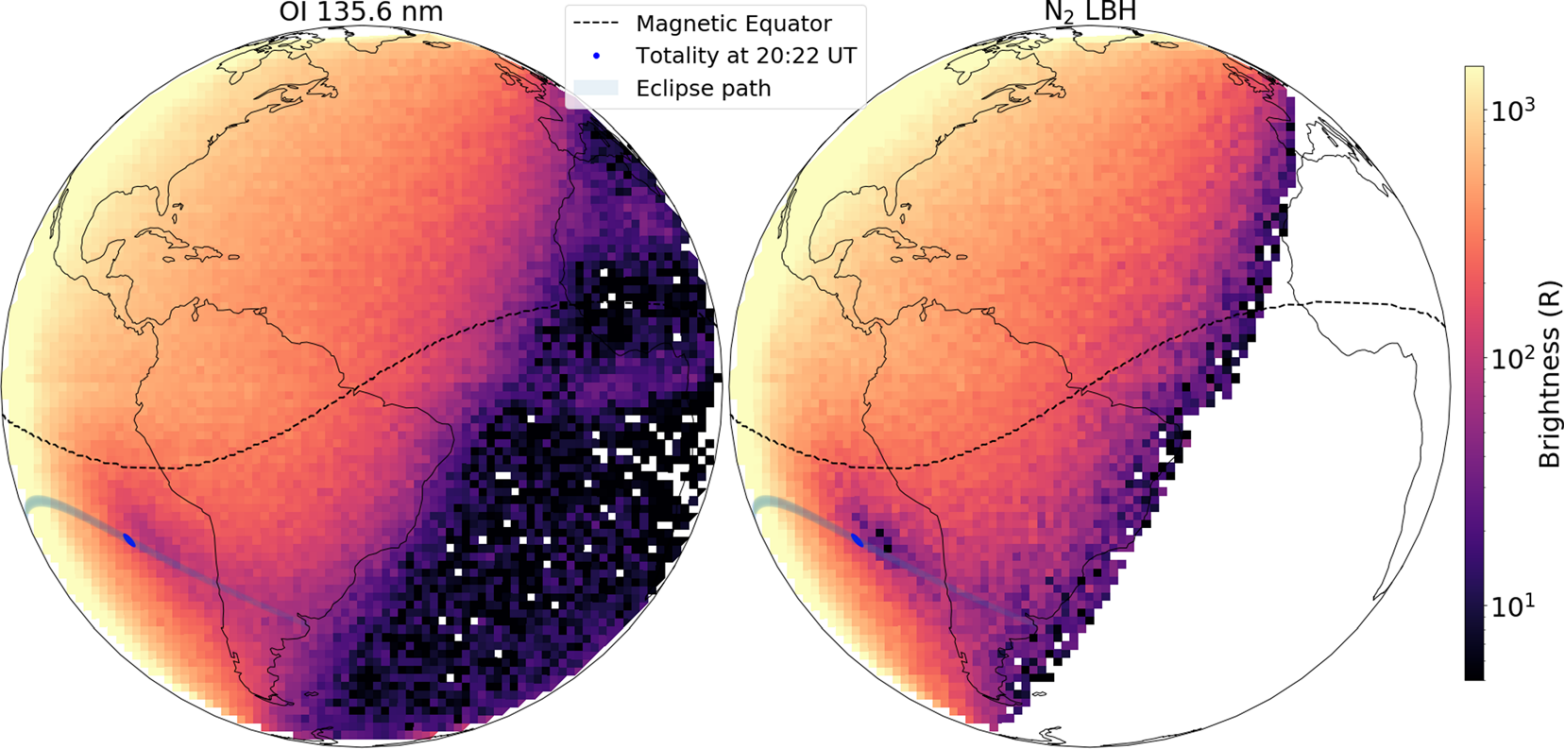
GOLD's imaging of the OI 135.6 nm (left) and N_2_ LBH (right) emission brightness (logarithmic scale) on 2 July 2019 during the total solar eclipse. The scans were made at a cadence of 30 min starting at 22:10 UT. Magnetic equator (dashed‐black), eclipse's path (shaded gray), totality at scan start on the Southern Hemisphere (blue‐dot), and the brightnesses are mapped to the continental outline. Notice that reduction in brightness is clear in both emissions very close to the totality.

The 2 July 2019 total solar eclipse started around 17 UT close to 36°S, 157°W and ended about 22 UT near 37°S, 57°W. Its greatest duration and extent occurred at ~19 UT, with a maximum totality of ~5 min (https://eclipse.gsfc.nasa.gov/). At a given time, the totality covered an area of ~1–2° latitude by 1–2° longitude. As the Moon's shadow moved toward the South American west coast from the west, it came within GOLD's field of view (FOV). Figure 2 shows GOLD's imaging of the eclipse in terms of 135.6 nm and the LBH brightnesses at 20:10 UT (scan start) on 2 July 2019. A reduction in both 135.6 nm and LBH brightnesses are seen near the totality. The observed reductions are a direct result of attenuated solar ultraviolet and X‐ray radiation within the eclipsed region. High‐energy solar radiation ionizes the upper atmosphere of the Earth creating energetic photoelectrons. The main production mechanism for 135.6 nm and the sole production mechanism for LBH are photoelectron impact on O and N_2_, respectively (Meier, 1991, and references therein). Thus, a reduction in the photoelectron flux within the eclipse's shadow leads to a reduction in the observed brightnesses of 135.6 nm and LBH emission. GOLD's observation shows that the brightness reduction in both 135.6 nm and LBH is immediate, suggesting rapid reduction in photoelectron flux due to eclipse's shadow (see Movies S1 and S2 in the supporting information). In addition to photoelectron impact, the 135.6 nm emission has an ~10% contribution from radiative recombination (RR) of O^+^ (Kil et al., 2013; Meier, 1991, and references therein). This is most likely the reason that the observed brightness reduction in LBH emission, which is produced entirely by photoelectron impact, is greater than the reduction in 135.6 nm emission.

## 3 Results and Discussion

**Figure 3:**
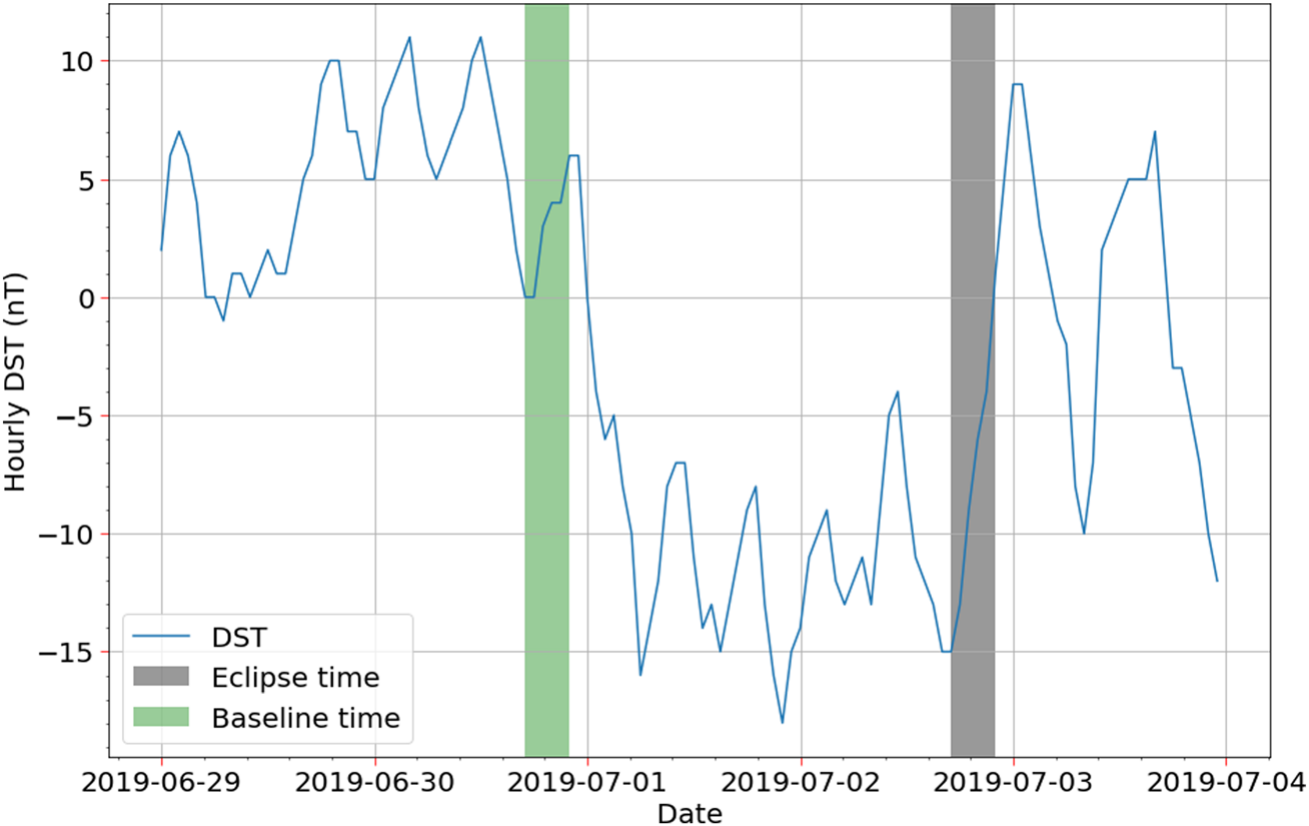
Hourly average of DST from 29 June to 3 July 2019. The duration of the eclipse and the 30 June 2019 baseline are represented by shaded black and green regions, respectively. Notice that the DST reaches approximately −17 nT on 1 July. DST data obtained from International Service for Geomagnetic Indices (ISGI, http://isgi.unistra.fr/geomagnetic_indices.php).

**Figure 4:**
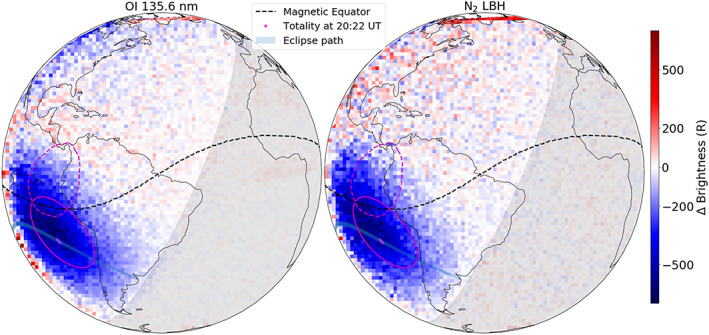
Difference in 135.6 nm and LBH brightness between the eclipse (2 July) and the baseline (30 June) days. In contrast to Figure 2, the full effect of the eclipse in terms of brightness depletion caused by both the umbral and penumbral shadow is seen. The totality location (magenta dot), magnetic equator (dashed black), and the nightside (lightly shaded region) are shown for reference. A region around the totality (solid magenta) and its magnetic conjugate (dashed magenta) are also shown to address potential conjugate effects. Notice a greater than 500 *R* depletion in both emissions compared to 30 June.

For quantification of the eclipse induced brightness reductions seen in Figure 2, day disk measurements from 30 June 2019 (one example being Figure 1), 2 days before the eclipse, are used as baselines. These scans were made identically as during the eclipse day (refer to section 2 for details). The solar flux remained effectively unchanged in between the observations: 67, 68, and 67 solar flux units on 30 June, 1 July, and 2 July, respectively (F10.7, obtained from NOAA). However, there was minor geomagnetic activity on 1 July and early part of 2 July, but not on the baseline day (Figure 3). Figure 4 shows differences in 135.6 nm and LBH brightnesses between the eclipse and baseline measurements at 20:10 UT. In addition to the umbral shadow (seen in Figure 2 as well), the penumbral shadow is also visible in Figure 4. Peak depletion of greater than 500 *R* is observed in both the 135.6 nm and the LBH emissions. Typical uncertainty in brightness for both 135.6 nm and LBH is ~50 *R* during the day and ~10–20 *R* (only for 135.6 nm) during the night (Figure S1).

Effects of photoelectrons from the sunlit conjugate hemisphere increasing the nighttime airglow have been reported recently (Solomon et al., 2020). For the 2017 eclipse, SAMI3 simulations predicted a reduction in electron density (including photoelectrons) at eclipse's conjugate hemisphere (Huba & Drob, 2017). To determine if GOLD observed any eclipse‐related conjugate effects, a region around totality and its magnetic conjugate are both shown in Figure 4 (solid and dashed magenta outlines, respectively). Since the eclipse shadow is near the magnetic equator, its conjugate region overlaps the shadow itself. Thus, there are no clear indications of any conjugate effects in the observations shown in Figure 4.

Figure 4 shows a reduction in 135.6 nm and an enhancement in LBH emissions at midlatitudes in the Northern Hemisphere relative to the 30 June 2019 baseline. This is likely because of enhanced auroral activity (at high latitudes) on 1 July, which persisted up to the eclipse time in Figure 4. While the geomagnetic conditions during GOLD's observations were mostly quiet (DST < −20 nT), there was minor activity on 1 July and early part of 2 July (Figure 3). Even minor auroral activity can cause joule heating at higher latitudes (Karan & Pallamraju, 2018; Gan et al., 2020b). High‐latitude auroral heating leads to atomic oxygen depleted meridional winds flowing toward midlatitudes. This wind flow reduces the 135.6 nm brightness and enhances the LBH emission brightness. To investigate this further, we look at ΣO/N_2_, which is a measure of compositional changes in the thermosphere (Correira et al., 2020; Evans et al., 1995; Strickland et al., 1995).

**Figure 5:**
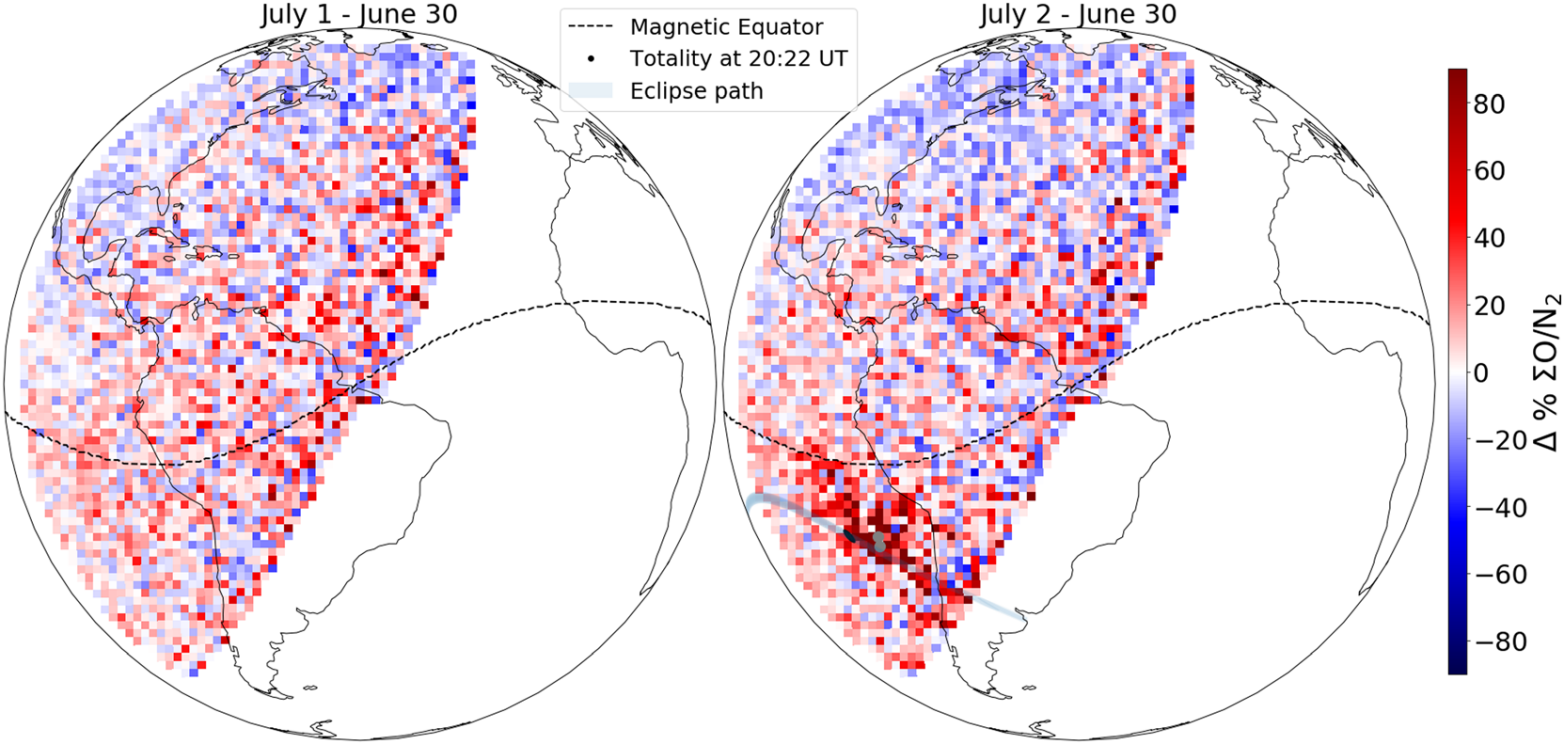
ΣO/N_2_ percentage changes on 1 (left) and 2 (right) July with respect to 30 June 2019 at 20:10 UT (scan start). Magnetic equator (dashed black) and continental outlines are shown for reference. For the eclipse day (right), the path of the eclipse and location of totality are also shown. Notice that ΣO/N_2_ ratio is significantly enhanced on the eclipse day (right) compared to the baseline near totality. The pixels grayed out (right, gray dots) close to totality are beyond the bounds of the ΣO/N_2_ retrieval algorithm.

Figure 5 shows ΣO/N_2_ percentage changes at 22:10 UT (scan start) on 1 and 2 July 2019 with respect to the baseline (30 June). The ΣO/N_2_ changes for both days are morphologically and quantitatively similar except within the eclipsed region. A significant ΣO/N_2_ increase relative to the baseline is observed around totality on the eclipse day, indicating enhanced atomic oxygen density. This is consistent with previous theoretical studies that predicted downwelling of atomic oxygen rich air into the totality region (Cnossen et al., 2019; Dang et al., 2018; Huba & Drob, 2017; Wang et al., 2019, and references therein). Both days also show depleted ΣO/N_2_ in the midlatitude Northern Hemisphere. As discussed in the last paragraph, this is most likely due to geomagnetic activity‐induced auroral joule heating and the associated circulation change.

Behind the eclipse's shadow, a greater increase in 135.6 nm emission is seen (but not in LBH) when compared to the increase seen in front of the shadow (Figure 4). This indicates an increase in ΣO/N_2_ ratio behind the eclipse's shadow in Figure 5. However, the derivation of ΣO/N_2_ depends on the solar zenith angle (SZA) and is less reliable at SZA > ~80° (Strickland et al., 1995). Any ΣO/N_2_ change behind the shadow is not obvious from Figure 5. Additionally, the derived ΣO/N_2_ uncertainty is a function of both the 135.6 nm and LBH uncertainties. This results in a lower signal‐to‐noise ratio (SNR) for ΣO/N_2_ when compared to the individual SNRs of 135.6 nm and LBH brightnesses. The ΣO/N_2_ uncertainty is the greatest near the totality because of the reduced SNR in both 135.6 nm and LBH emissions, plus the O^+^ RR contribution in 135.6 nm (see previous section). The nighttime 135.6 nm brightness during the eclipse time is ~20–30 *R* near the Equatorial Ionization Anomaly (EIA) and gives an upper limit on the RR contribution (see Figure S2). Based on TEC measurement (Figure S3) near the eclipse path (but away from EIA) and using the empirical algorithm that converts the square of TEC to RR brightness (Figures 11 and 12 in Rajesh et al., 2011), the O^+^ RR is <5 *R*. Assuming a maximum mean RR contributed 135.6 nm brightness of 25 R (based on EIA strength) and a minimum mean dayglow 135.6 nm brightness of 80 R near the totality (see Figure S4), the corresponding maximum ΣO/N_2_ systematic error is estimated to be ~35% using standard error propagation techniques. The pixels that are grayed out (Figure 5, right, gray dots) are outside the bounds of the ΣO/N_2_ retrieval algorithm and thus are not reported.

### 3.1 Special Observation Mode

**Figure 6:**
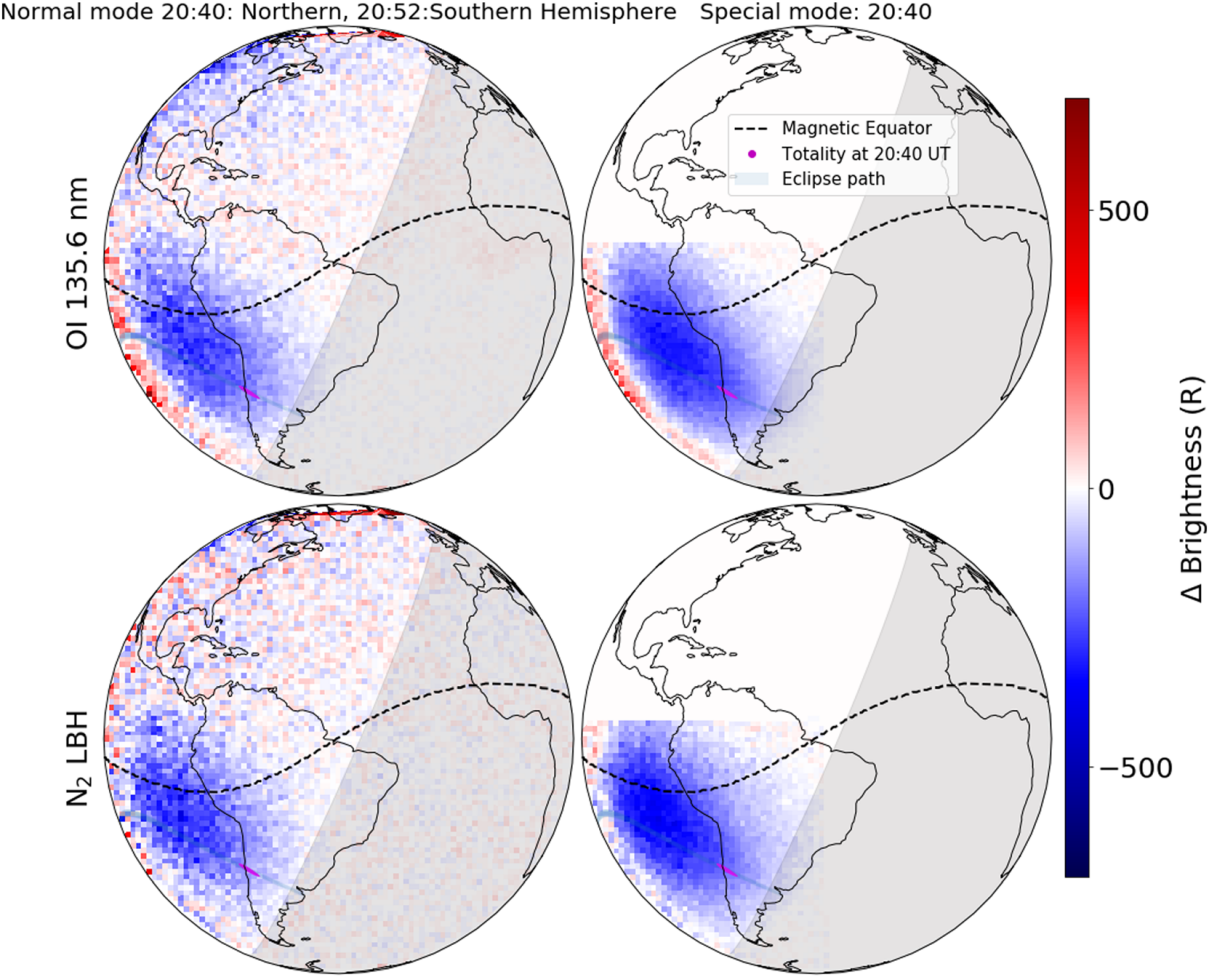
Comparison between the full‐disk (left) and the “special” (right) observation of eclipse induced brightness change (compared to 30 June baseline) at the scan start time of 20:40 UT on 2 July 2019. Notice that the brightness changes are quantitatively similar for both, but the special mode is less noisy. Also notice the slight offset in the eclipse shadow between the full‐disk and the special mode. This offset is because the two scan modes start their scan at different geographic locations and their dwell times are also different.

As the eclipse was confined to the southwest corner of GOLD instrument's FOV, we used its second channel to only observe that specific region. This “special” observation's cadence was kept identical to the full‐disk observation (~30 min). But as the scans were confined to the southwest corner, it permitted four times longer integration. This resulted in SNR that was twice as large as full‐disk observations. In preparation for the special eclipse observation, identical measurements were first made on 30 June and used as baselines. Comparisons between the baseline‐subtracted full‐disk and special observation brightness changes (for 135.6 nm and LBH) at 20:40 UT (scan start) are shown in Figure 6. It is seen that, although the special observation has a higher spatial resolution and lower noise level, the quantitative changes in brightness are almost identical to the full‐disk observations. We also see a slight difference in the location of the eclipse shadow between the full‐disk and the special observations. This is because the scan‐start location and the scan step taken by the two channels (for full‐disk and special observation) are different. The typical uncertainty for the special observation daytime brightnesses is ~20–30 *R* (Figure S5).

**Figure 7:**
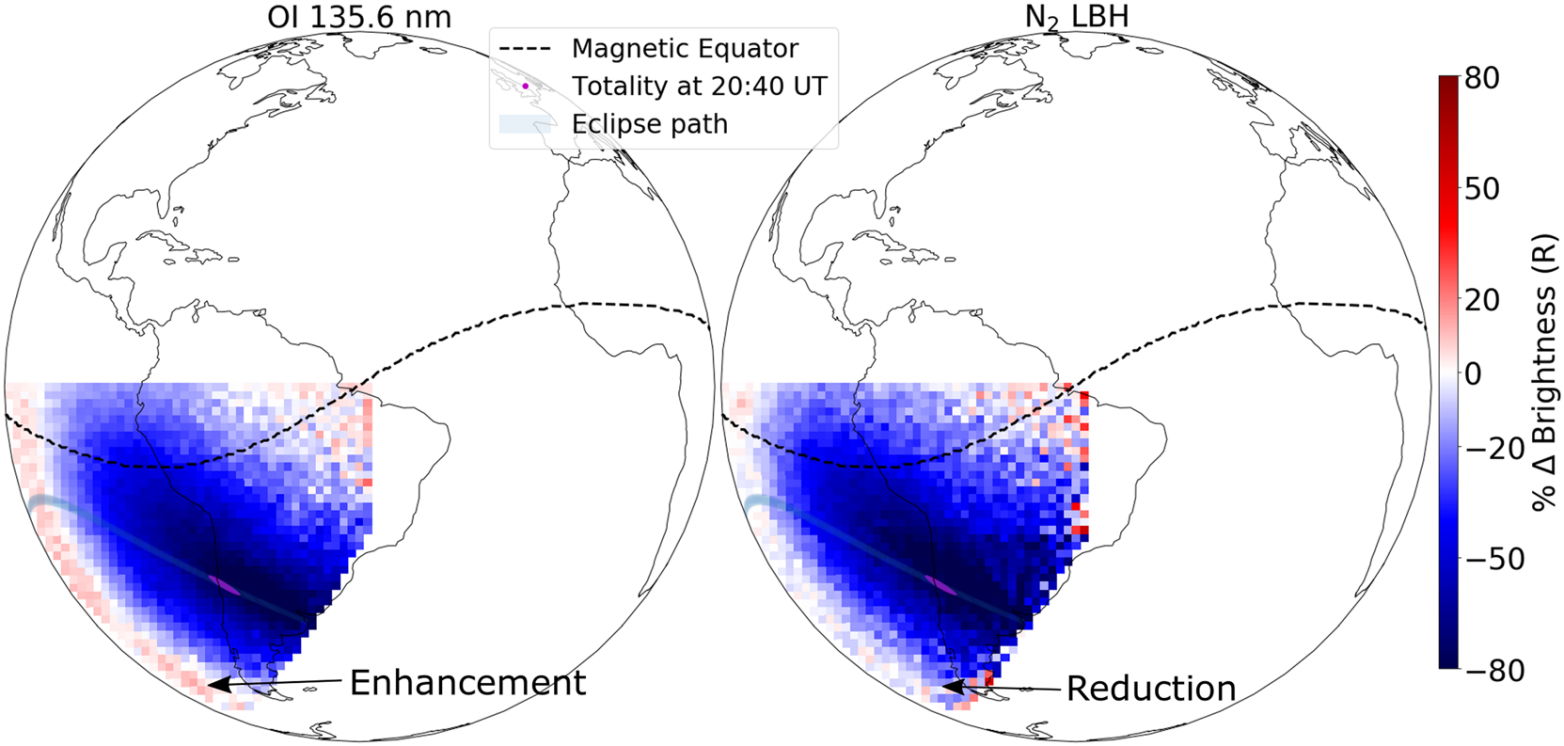
GOLD's “special” observation of percentage change in 135.6 nm and LBH brightnesses at 20:40 UT (scan start) on 2 July 2019 compared to the baseline. Notice >80% depletion in both brightnesses near totality. Also notice that the 135.6 nm is enhanced and the LBH emission is reduced behind the eclipse.

Percentage difference in brightness with respect to the baseline measurement (special observation) is presented in Figure 7. A brightness reduction of greater than 80% is observed in both 135.6 and LBH emissions close to the totality. Behind the eclipses' shadow, an increase in 135.6 nm (~15%) and a slight decrease (>10%) in LBH brightness is observed relative to the baseline.

**Figure 8:**
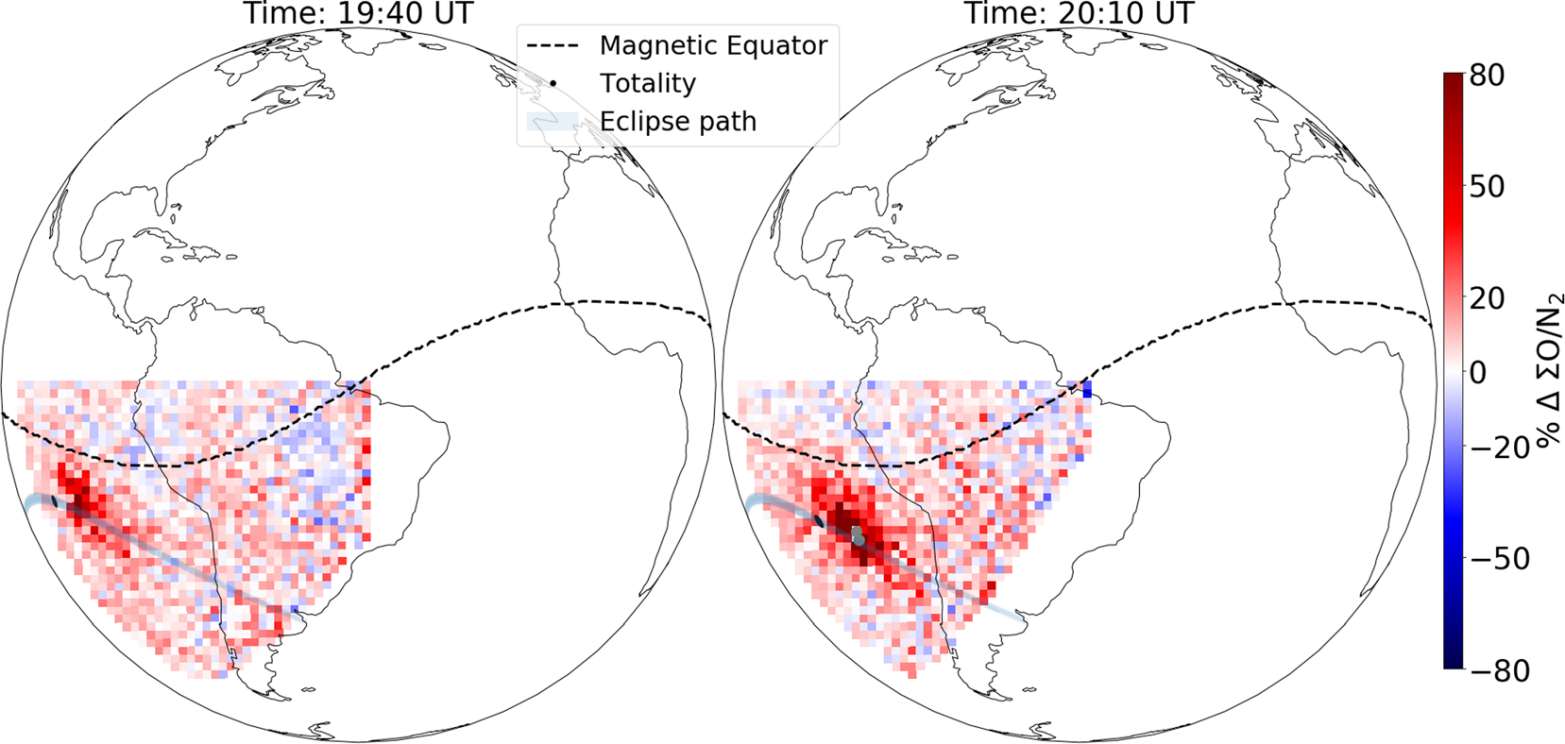
Special observation mode: ΣO/N_2_ percentage change (with respect to 30 June) at 19:40 and 20:10 UT (scan start) on 2 July 2019. The magnetic equator (dashed‐black), eclipse's path (shaded gray) and the continental outlines are shown for reference. Notice ~80% enhancement in ΣO/N_2_ around the totality (black dot) for both times. The pixels grayed out (right, gray dots) close to the totality are beyond the bounds of the ΣO/N_2_ retrieval algorithm.

For better quantification of compositional changes, ΣO/N_2_ percentage change (special observation) is shown in Figure 8 at 19:40 and 20:10 UT (scan start). Significant ΣO/N_2_ enhancement (~80%) is observed at both times around the totality. As discussed in the previous section, this strongly suggests that the eclipse induced major compositional changes.

**Figure 9:**
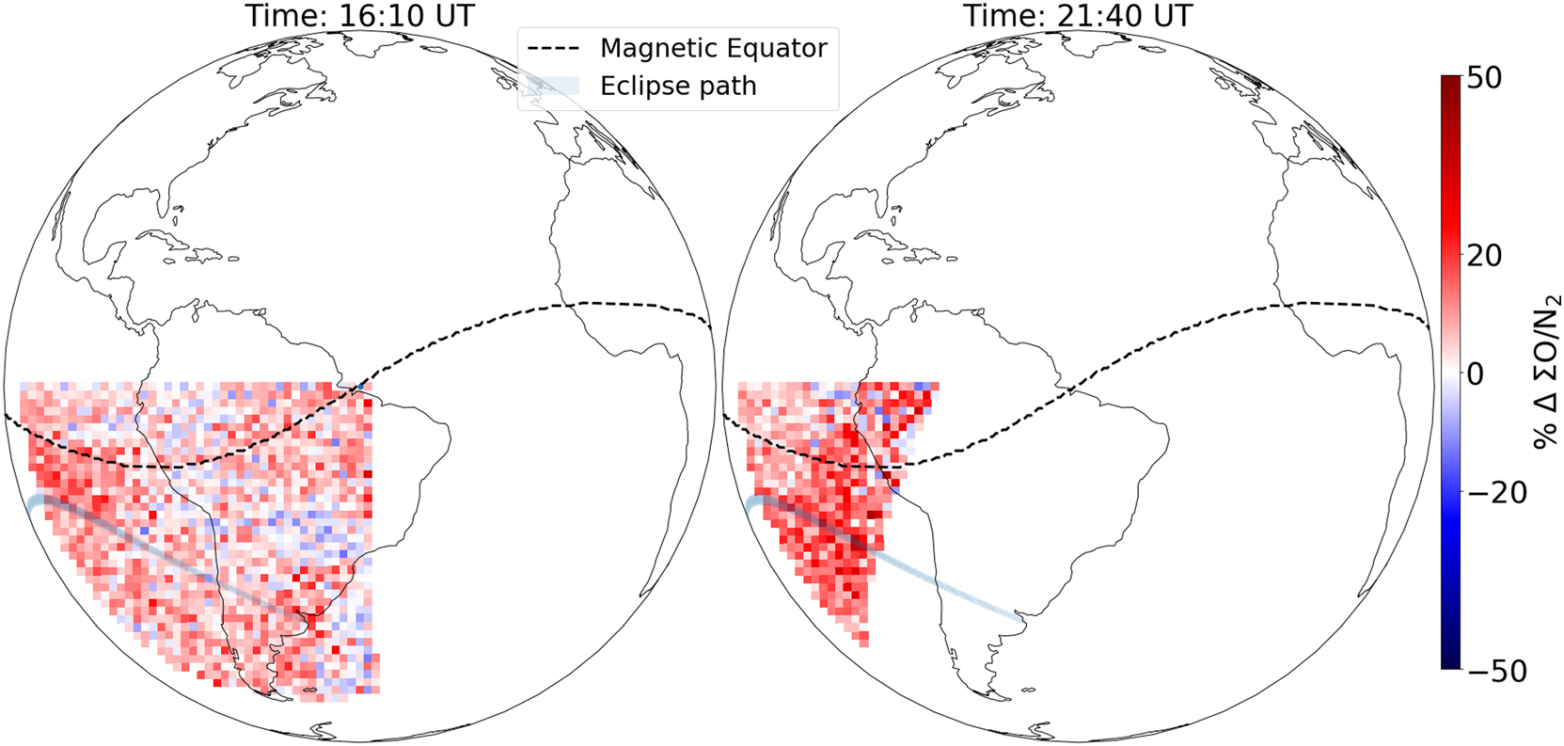
Special observation mode: ΣO/N_2_ percentage change (with respect to 30 June) before the eclipse at 16:10 and after the eclipse at 20:10 UT (scan start) on 2 July 2019. The magnetic equator (dashed‐black), eclipse's path (shaded gray), and changes in ΣO/N_2_ are mapped to the continental outline. Notice a slight enhancement in the ΣO/N_2_ percentage difference after the eclipse, near the eclipse's path.

**Figure 10:**
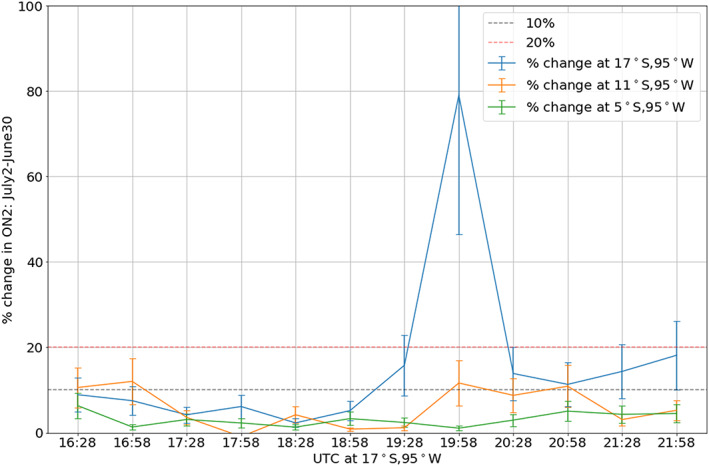
Average (in 2° by 2° latitude and longitude) ΣO/N_2_ percentage changes (with respect to 30 June) at three different geographic location as a function of scan time. Notice that for 17°S, 95°W (blue line), there is enhanced change after the eclipse (19:58 UT), compared to before the eclipse. This is not apparent at other latitudes.

Any effect of the eclipse behind the eclipse shadow was not obvious from Figure 5. Thus, to investigate this potential after effect of the eclipse, ΣO/N_2_ percentage change before the eclipse (16:10 UT scan start) and after the eclipse (21:40 UT scan start) is presented in Figure 9. While the ΣO/N_2_ before the eclipse was enhanced too (~10%) compared to 30 June, the enhancement after the eclipse was higher (~20%). To establish the statistical significance of the enhancement seen after the eclipse, average (in 2° by 2° latitude and longitude) ΣO/N_2_ percentage change at one location (17°S, 95°W, in the path of totality) is shown as a function of scan time in Figure 10 (light blue curve). Average ΣO/N_2_ percentage change at two other latitudes (but same longitude: 95°W), 11°S and 5°S, is also shown in Figure 10. The 80% enhancement centered at 19:58 UT around 17°S (blue line, in the path of the totality) is within the totality. At the same location (17°S), a slightly higher (~20%) enhancement is seen centered at 21:58 UT (Figure 10) compared to the enhancement before the eclipse (~10% at 16:28 UT). For the other two latitudes, any such enhancement is not obvious. The average ΣO/N_2_ percentage change (in time) at 17°S for times before the eclipse is 5.7 ± 2.7%. For the times after the eclipse, it is 13.2 ± 6%. Thus, we conclude that a slight enhancement in ΣO/N_2_ (~7%) remained even after the eclipse, near the eclipse's path.

## 4 Conclusions

In this paper, we have presented the first global‐scale synoptic imaging of the 2 July 2019 solar eclipse's thermospheric effects by the GOLD instrument aboard the SES‐14 satellite in a geostationary orbit. A significant reduction (>80%) in 135.6 nm and LBH emissions brightnesses were observed near totality. This was a direct result of a decrease in photoelectron flux caused by the eclipse. Within the totality, a significant increase in ΣO/N_2_ (~80%) was also observed. Previous theoretical studies have predicted this increase as a consequence of atomic oxygen rich air downwelling into the region associated with the cooling and wind convergence caused by the eclipse. In addition, a slight ΣO/N_2_ enhancement (~7%) was also seen after the eclipse, close to the eclipse's path. These results suggest the eclipse caused compositional changes in the thermosphere.

Eclipse‐induced changes represent a unique, rapidly evolving driver of the IT system. The temporal response of the measured parameters (e.g., airglow brightnesses) to the eclipse‐impulse could help us better quantify and/or validate different production and decay rates of important upper atmospheric photo‐chemical processes. These cannot easily be determined during the sunlit, night, or day‐night transition conditions. Thus, future detailed data‐model comparison between GOLD's eclipse observations (plus other supplementary measurements) with IT models could allow us to test and expand our understanding of IT system behavior.

## Supporting information

Supporting Information Data S1

Movie S1

Movie S2

Figure S1

Figure S2

Figure S3

Figure S4

Figure S5

## Data Availability

GOLD L1C and L2 data used for this study can be accessed at the GOLD Science Data Center (http://gold.cs.ucf.edu/search/) and at NASA's Space Physics Data Facility (https://spdf.gsfc.nasa.gov).
