# Supplementary material for: First Global‐Scale Synoptic Imaging of Solar Eclipse Effects in the Thermosphere
Source: J Geophys Res Space Phys. 2020 Sep 18;125(9):e2020JA027789. doi: 10.1029/2020JA027789 (PMC7685169; doi:10.1029/2020JA027789)
Supplement: Supplementary file 1 — Supporting Information Data S1 [file JGRA-125-e2020JA027789-s001.docx]

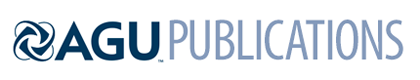


JGR: Space Physics

Supporting Information for

**First Global-scale Synoptic Imaging of a Solar Eclipse Effects in the Thermosphere.**

Saurav Aryal^1^, J. S. Evans^2^, John Correira^2^, Alan G. Burns^3^, Wenbin Wang^3^, Stanley C. Solomon^3^, Fazlul I. Laskar^1^, William E. McClintock^1^ and Richard W. Eastes^1^, Tong Dang^4^, Jiuhou Lei^4^, Huixin Liu^5^, Geonhwa Jee^6^

^1^Labrotary for Atmospheric and Space Physics, University of Colorado, Boulder.

^2^Computational Physics Inc.

^3^High Altitude Observatory, National Center for Atmospheric Research.

^4^University of Science and Technology of China

^5^Kyushu University

^6^Division of Polar Climate Sciences, Korea Polar Research Institute, Incheon, Republic of Korea

Corresponding author: Saurav Aryal ([saurav.aryal@lasp.colorado.edu)](mailto:saurav.aryal@lasp.colorado.edu))

**Contents of this file**

Figures S1 to S5

**Additional Supporting Information (Files uploaded separately)**

Captions for Movies S1 to S2

**Introduction**

The video files are provided in .avi format.


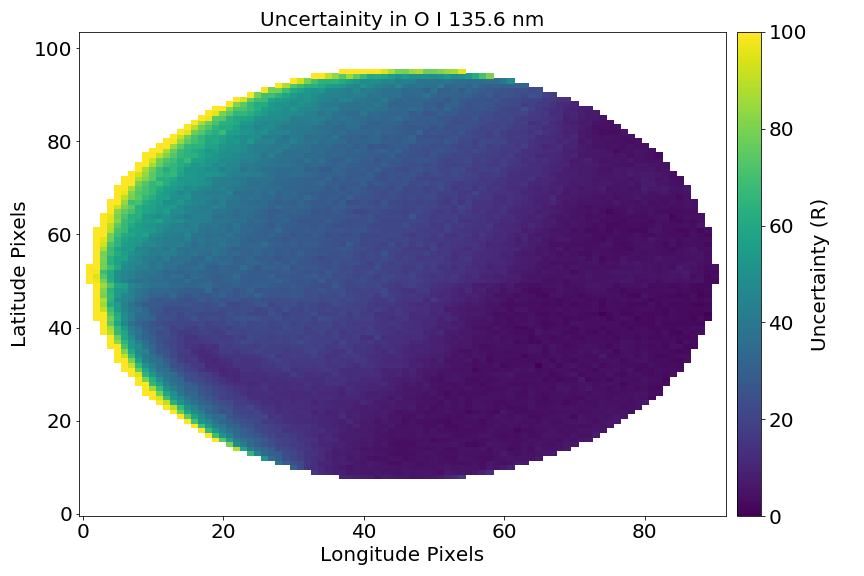
Figure S1. Typical full-disk brightness uncertainty in 135.6 nm brightness during one of the eclipse time (19:40 UT, July 2, 2019). LBH brightnesses have similar day-time uncertainty.


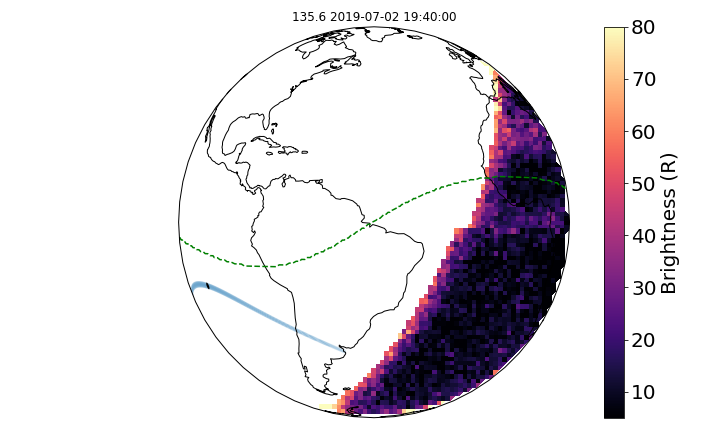


Figure S2. Night-side EIA strength at 19:40 UT during the July 2, 2019 eclipse. Notice the peak 135.6 nm EIA brightness of ~ 20-30 R (away from the terminator). The magnetic equator (dashed-green) and the continental outlines are shown for reference.


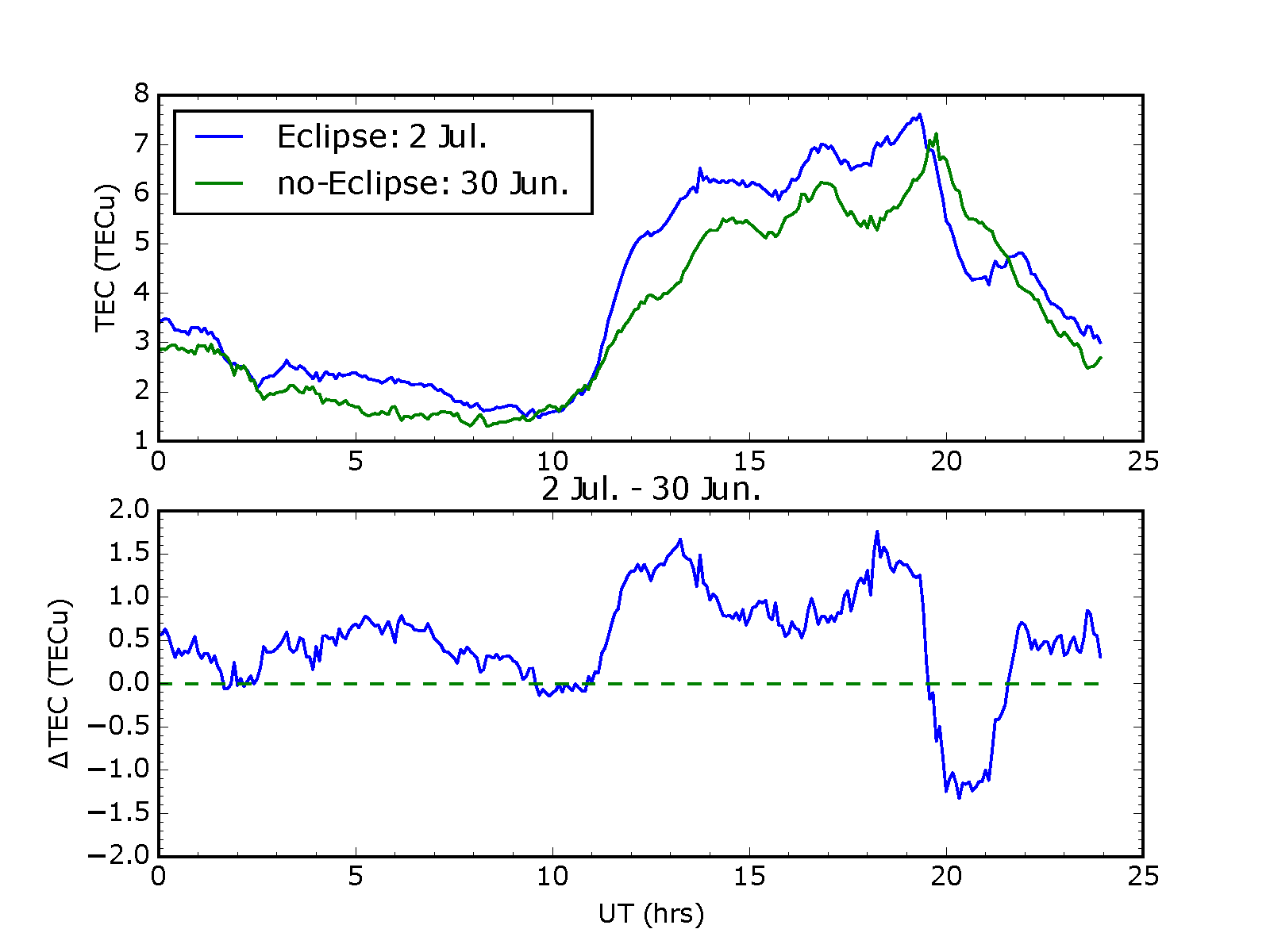


Figure S3. : Comparison of TEC between the Eclipse day and June 30 at 36º S, 64º W, ~3º latitude away from the totality. The depletion in TEC (bottom) around 20 UT is due to the eclipse.


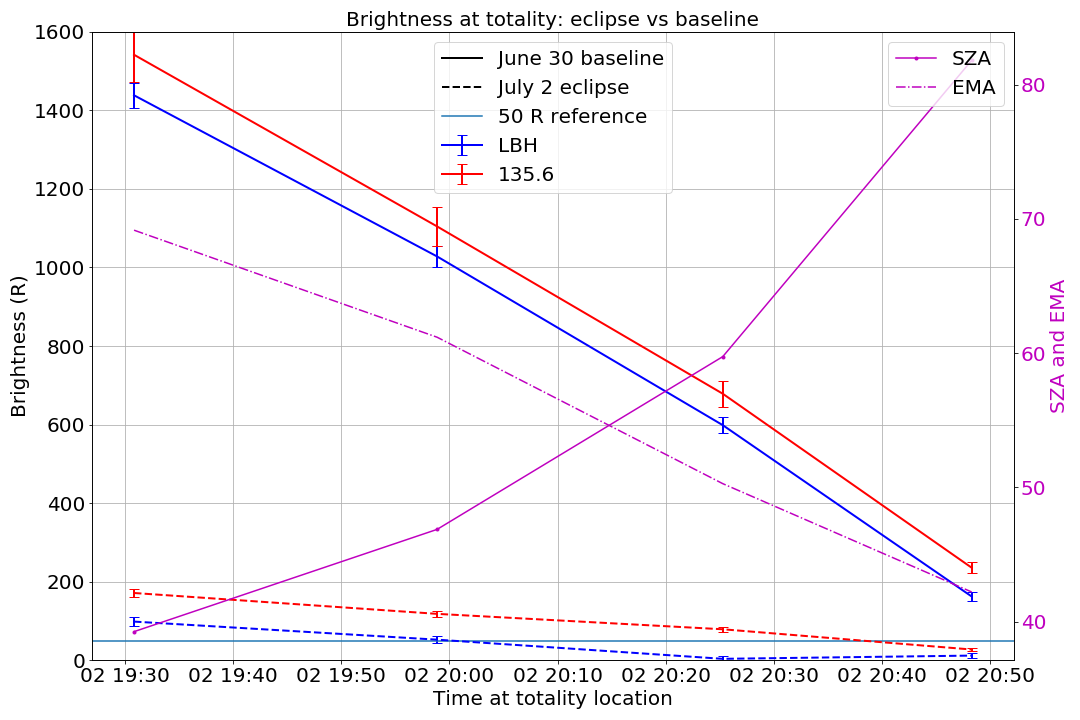


Figure S4. : Comparison of average 135.6 nm and LBH brightnesses at totality location between the eclipse day (July 2) and June 30, 2019 for the four scans where the totality is in GOLD’s FOV. Solar zenith angle (SZA) and emission angle (EMA) at the totality location are also shown for reference.


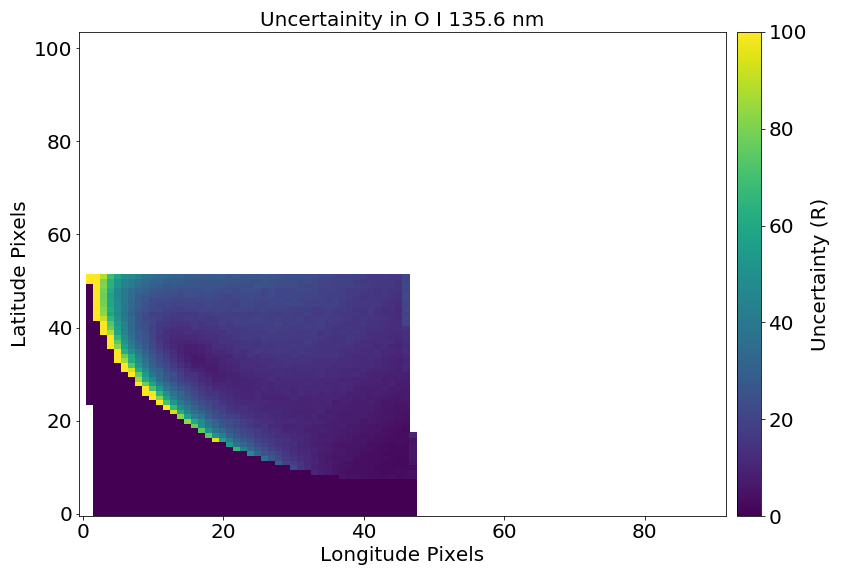
Figure S5. : Typical Uncertainty in 135.6 nm brightness during one of the eclipse time (19:40 UT, July 2, 2019): Special observations. LBH brightnesses have similar daytime uncertainities.

Movie S1. All the full-disk imaging of GOLD’s eclipse observation as a video. The video shows 135.6 nm and LBH brightnesses on the eclipse day (July 2, 2019).

Movie S2. All the full-disk difference imaging of GOLD’s eclipse observation. The images are shown as eclipse day (July 2, 2019) brightness change with respect to June 30 (baseline).
