# Supplementary figures and images for: First Global‐Scale Synoptic Imaging of Solar Eclipse Effects in the Thermosphere
Source: J Geophys Res Space Phys. 2020 Sep 18;125(9):e2020JA027789. doi: 10.1029/2020JA027789 (PMC7685169; doi:10.1029/2020JA027789)

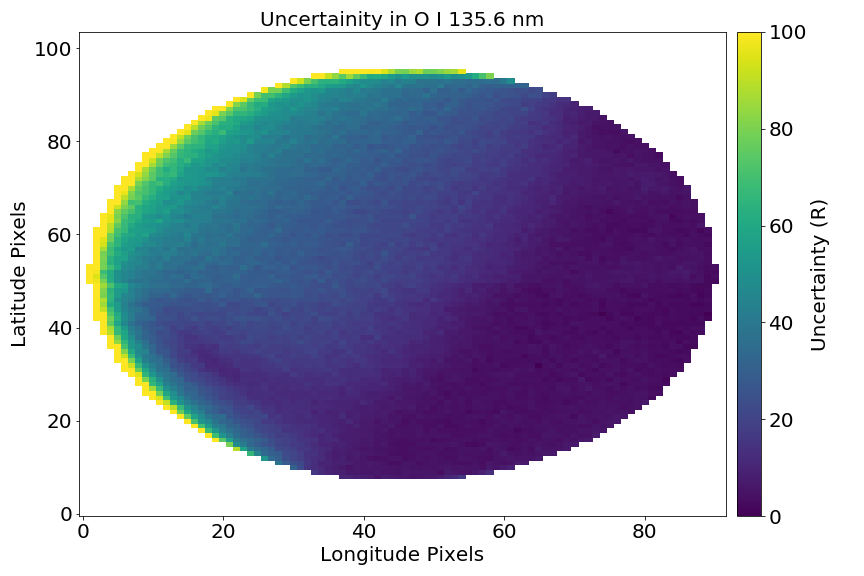

Supplement: Supplementary file 4 — Figure S1 [file JGRA-125-e2020JA027789-s004.png]

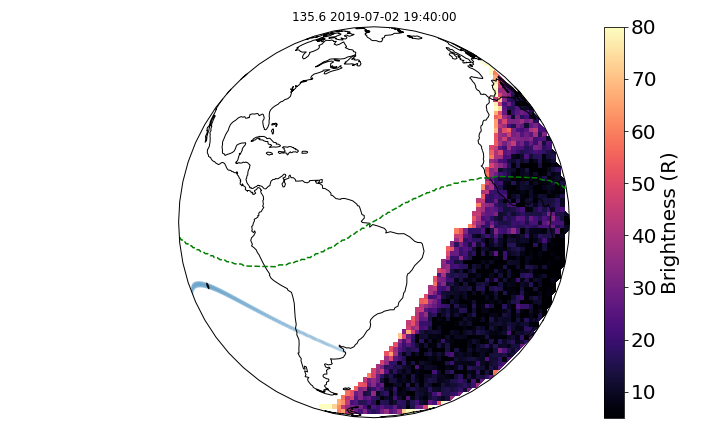

Supplement: Supplementary file 5 — Figure S2 [file JGRA-125-e2020JA027789-s005.png]

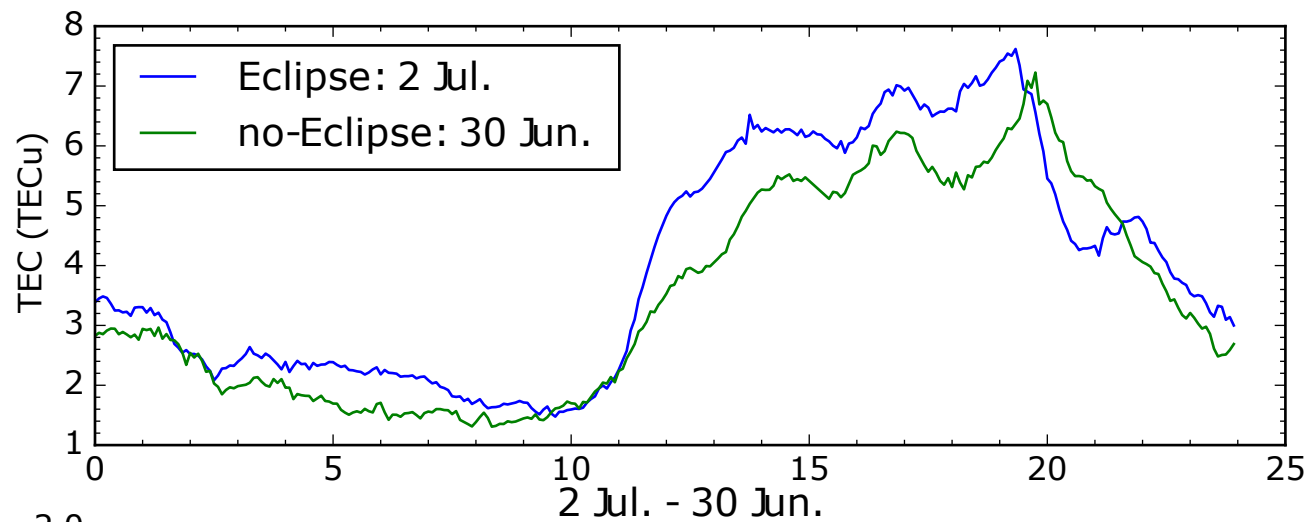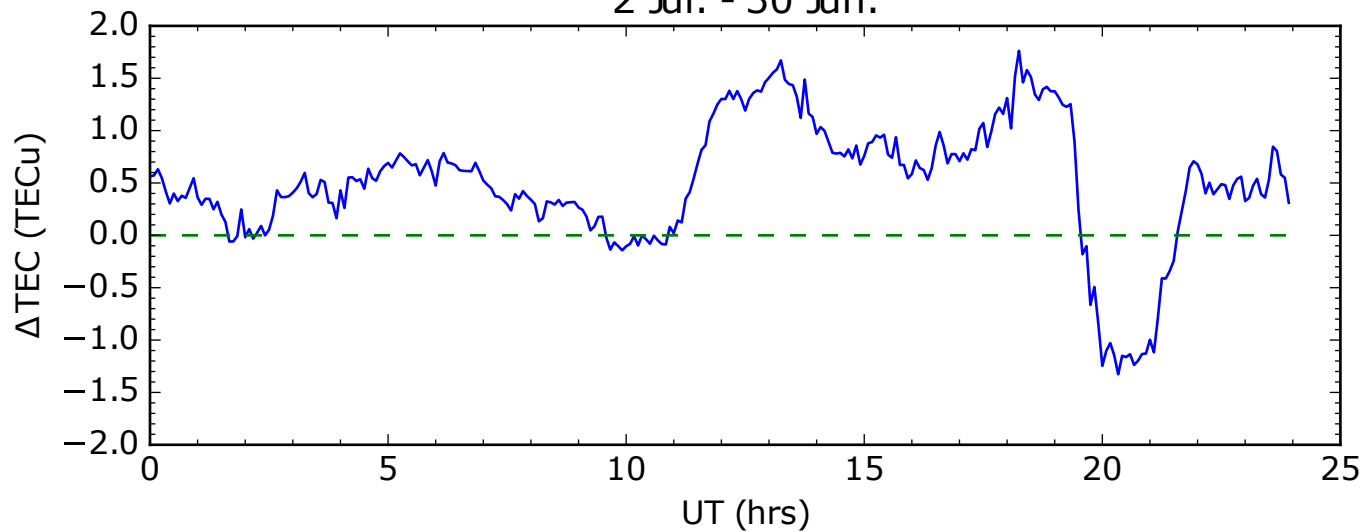

Supplement: Supplementary file 6 — Figure S3 [file JGRA-125-e2020JA027789-s006.pdf]

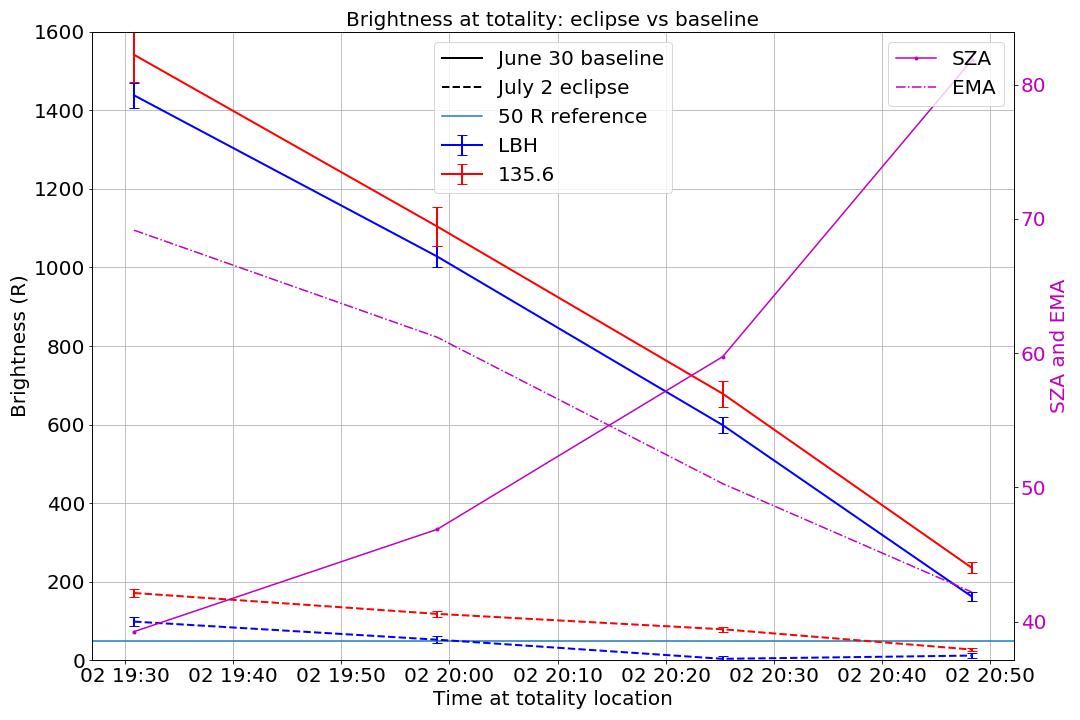

Supplement: Supplementary file 7 — Figure S4 [file JGRA-125-e2020JA027789-s007.png]

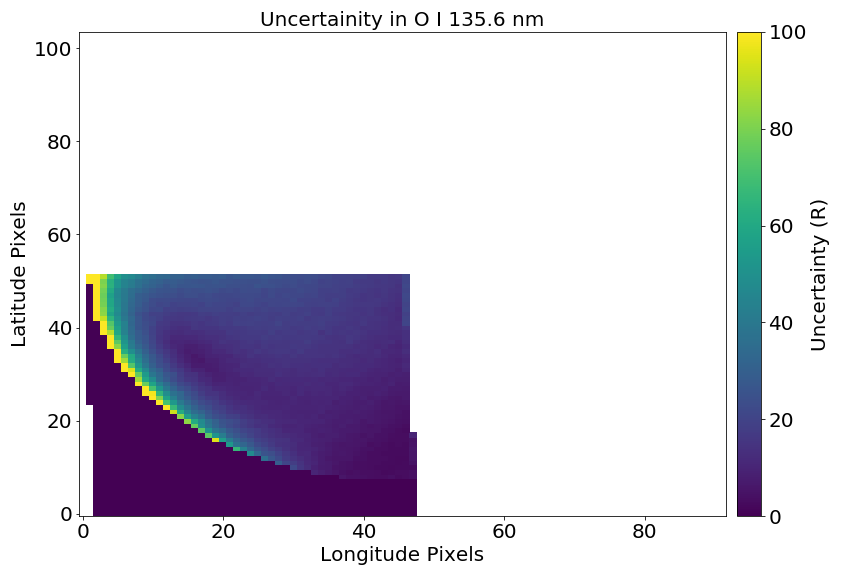

Supplement: Supplementary file 8 — Figure S5 [file JGRA-125-e2020JA027789-s008.png]
